# Microevolution and phylogenomic study of Respiratory Syncytial Virus type A

**DOI:** 10.1371/journal.pone.0319437

**Published:** 2025-02-25

**Authors:** Ashfaq Ahmad, Sidra Majaz, Aamir Saeed, Shumaila Noreen, Muhammad Abbas, Bilal Khan, Hamid Ur Rahman, Faisal Nouroz, Yingqiu Xie, Abdur Rashid, Atta Ur Rehman

**Affiliations:** 1 Department of Bioinformatics, Faculty of Natural and Computational Sciences, Hazara University, Mansehra, Khyber Pakhtunkhwa, Pakistan; 2 Department of Zoology, Faculty of Biological and Health Sciences, Hazara University, Mansehra, Khyber Pakhtunkhwa, Pakistan; 3 Department of Urology, Pakistan Institute of Medical Sciences, Islamabad, Pakistan; 4 Department of Pediatrics, Tehsil Headquarter Hospital (THQ), Dargai, Malakand, Khyber Pakhtunkhwa, Pakistan; 5 Department of Biology, School of Sciences and Humanities, Nazarbayev University, Astana, Kazakhstan; 6 Government Degree College Ara Khel, F.R Kohat, Higher Education Department, Government of Khyber Pakhtunkhwa, Kohat, Khyber Pakhtunkhwa, Pakistan; Taif University, SAUDI ARABIA

## Abstract

Communal respiratory syncytial virus (RSV) causes mild to severe illnesses, predominantly in older adults, or people with certain chronic medical conditions, and in children. Symptoms may include rhinorrhea, cough, fever, and dyspnea. In most cases, the infection is mild and resolves on its own, but in some cases, it can lead to more serious illness such as bronchiolitis or pneumonia. The RSV genome codes for ten proteins, NS1, NS2, N, P, M, SH, G, F, M2 and L. We aimed to identify the RSV geographical transmission pattern based on parsimony and investigate hotspot regions across the complete RSV genomes. We employed Viral Evolutionary Network Analysis System on full-length available RSV genomes and with HyPhy for elucidating type of selection pressure. These results indicated that RSV strains circulating in South and North America are not mixed to the European samples, however, genomes reported from Australia are the direct decedents of European samples. Samples reported from the United Kingdom exhibited significant diversity, spanning almost every cluster. This report provides a complete mutational analysis of all the individual RSV genes, and particularly the 31 hotspot substituting regions circulating across the globe in RSV type A samples. Further, protein G and L displayed higher level of codons experienced positive selection. This analysis of RSV type A highlights mutational frequencies across the whole genome, offering valuable insights for epidemiological control and drug development.

## Introduction

Viruses have long been a significant threat to humanity and have recently garnered increased attention due to their potential to cause large-scale pandemics, as evidenced from COVID-19 [1]. Respiratory syncytial virus (RSV), also known as human respiratory syncytial virus (hRSV) contributes to the infection of respiratory tract [2]. Initially, RSV was isolated from chimpanzee in 1956 and was also simultaneously recovered from human infants with severe lower tract respiratory disease [3]. In clinical manifestations, RSV is linked to mild upper respiratory tract illness (URTI) or otitis media to severe and potentially life-threatening lower respiratory tract involvement (LRTI). The most prevalent form of LRTI in RSV-infected newborns is bronchiolitis, however there are reports indicating pneumonia and croup. Approximately, in infants and young children ∼ 15–50% of the lower airways were found affected in primary RSV infection that results in hospitalization and higher mortality [4,5]. Apart from supportive care like fluid intake and rest, so far there is no specific treatment for RSV infection [6].

RSV is a non-segmented negative-sense single-stranded enveloped RNA virus that belongs to the family of *Paramyxoviridae*, genus *Pneumovirus*, and subfamily *Pneumovirinae*. The complete genome of RSV encodes ten proteins, i.e., NS1, NS2, N, P, M, SH, G, F, M2 and L. Among them, M and M2 are envelope proteins, while the fusion (F) and G are glycoproteins, and SH is a small hydrophobic protein. Besides, five structural and non-structural proteins are coded by the RSV genome includingthe large (L) protein, phosphoprotein (P), nucleocapsid (N), and non-structural proteins 1 and 2 (NS1 and NS2). Among them, protein G and F are important in host cell attachment, fusion and cellular entry [5,7–9].

The RSV has been classified into two subtypes A and B which further includes strains like GA1 - GA7, SAA1, NA1 - NA4 and ON1 (RSV-A), and GB1 - GB4, SAB1 - SAB4, URU1 - URU2, BA1 - BA10, BA - C and THB (RSV-B) [10,11]. There are reports discussing RSV mutations in different lineages [12–14], however we did not find extensive analysis for the detection of hotspot regions across the whole RSV genome. Here we are reporting for the first time all the mutational frequencies, hotspot regions and transmission of RSV subtype A. These analyses highlight a wider view of RSV transmission across different geological zones that could aid in predicting the oncoming pandemic and vaccine development. Besides transmission, this report provides all the observed mutations in genome coding regions and particularly the hotspot nucleotide sites prone to mutations in individual genes of RSV.

## Methodology

All the sequences used in these analyses were collected from the NCBI Virus database [15], where we selected taxonomic identification or taxid 208893, and spotted 11956 nucleotide sequences. Specific filters were applied for genome completeness, and only those genomes were considered, which were present in with sequence tag of complete, and thus the final dataset we found contained 871 sequences. The whole dataset was exported to MAFFT for whole genome alignment [16]. Next, the aligned dataset was fed to the viral genome evolutionary analysis system (VENAS) for further analyses [17].

### Calculation for effective parsimony-informative site (ePIS) and network construction

To calculate ePIS, we followed a rule that the site will be considered parsimony-informative if it contains a minimum of two types of nucleotides in the aligned data, and at least two sites of them should occur with a minimum nucleotide frequency of two. Besides, the ePIS were considered effective only in the case that the site must contain unambiguous bases ≥80% of the total genomes. Keeping the above rules, 871 genomes were automatically reduced to 474 genomes were found satisfactory, and thus all the remaining analyses were carried out on the dataset containing 474 genomes. The derived ePIS results were used to classify all the genomes in haplotypes, and for this reason sequences containing similar ePIS were grouped onto the similar nodes and vice versa. All the haplotypes were next visualized by Gephi, where further haplotype networking was performed, i.e., community detection, graph rendering followed by visual inspection.

### Mutational analysis

All the mutational analyses for individual genes were performed by NGS analysis package BioAider [18]. Nucleotide sequences for all the RSV individual genes were extracted from the aligned dataset of 474 genes. To classify the nature of identified mutations, each gene of RSV was handled separately, and the gaps were effectively removed. Next, we re-aligned these separated genes datasets through codon alignment tool, a BioAider functionality. Codon based alignment of each gene dataset was further analyzed for mutations and its classification. The complete reports for individual genes mutations can be found in S1 Table. To identify hotspot regions across the coding regions of the genomes, we applied a criterion that focused solely on non-synonymous substitutions. These substitutions were included only if they resulted in changes to amino acid properties and were observed in more than 200 samples. The auxiliary art work was drawn by illustrator for the visualization of biological sequences (IBS) and Paint.net [19].

### Evolutionary analysis using HyPhy SLAC

To investigate the evolutionary dynamics of the RSV genes, we performed a Sitewise Likelihood Ancestral Counting (SLAC) analysis using the HyPhy software suite (version 2.5.0) [20]. SLAC is a robust method for detecting site-specific selection pressures by comparing the rates of nonsynonymous (dN) and synonymous (dS) substitutions across a codon alignment. An alignment in FASTA and tree files in Newick was prepared in MAFFT for each gene. The tree was constructed using a maximum likelihood approach with appropriate substitution models. The SLAC analyses were conducted and results were retrievd in. JSON format, which includes detailed statistics for each codon site, such as dN, dS, ω, and p-values for selection tests.

The SLAC algorithm calculates the dN/dS ratio for each codon site in the alignment. Sites with ω > 1 are indicative of positive selection, while sites with ω < 1 suggest purifying selection. Sites with ω ≈ 1 are evolving neutrally.

### Nucleotide diversity, Shannon entropy and Tiajima’s D calculations

To analyze the evolutionary dynamics of the RSV genes, we employed a computational pipeline implemented in Python where we employed libraries from biopython. The alignment files in FASTA format were processed to calculate three key evolutionary metrics: Shannon entropy, nucleotide diversity (π), and Tajima’s D. Shannon entropy was computed for each position in the alignment to quantify site-specific variability, while nucleotide diversity provided a measure of average genetic variation across the entire sequence. Tajima’s D was calculated to assess deviations from neutral evolution, with negative values indicating potential population expansion or positive selection, and positive values suggesting balancing selection or population structure. The results, including position-specific entropy, nucleotide diversity, and Tajima’s D, were compiled into a CSV file for further analysis.

## Results

We retrieved the complete nucleotide sequence dataset for RSV type A (taxid. 1439707) from the NCBI Virus database. By December 2022, it contained 1166 nucleotide records, including 871 complete genomes. After initial filtration and name tagging that includes genome ID, reported year and country, we applied viral genome evolutionary analysis system (VENAS) [17] and analyzed the evolutionary relationship between different genomes or RSV strains. Among them the earlier reported genome U39662.1 in 1997 was considered as a reference. Based on the effective parsimony informative sites (ePIS), and removal of redundant genome sequences, VENAS picked 474 genomes for further analyses. The final genome dataset contains sequences from the USA (2014, 2017, 2019, and 2021), Brazil (2021), Kenya (2021), Philippines (2017), Jordan (2018), Thailand (2019), Netherland (2021), Australia (2020, and 2022), China (2018), UK (2021), Spain (2021), Germany (2020), and four Unknown sequences (2022). In total our dataset 96 complete genomes samples from the USA, 26 (Spain), 38 (Brazil), 44 (Australia), 50 (UK), 62 (Netherland), 08 (Thailand), and fewer were found from other countries.

### RSV transmission distribution on the country’s scale

**Fig 1:**
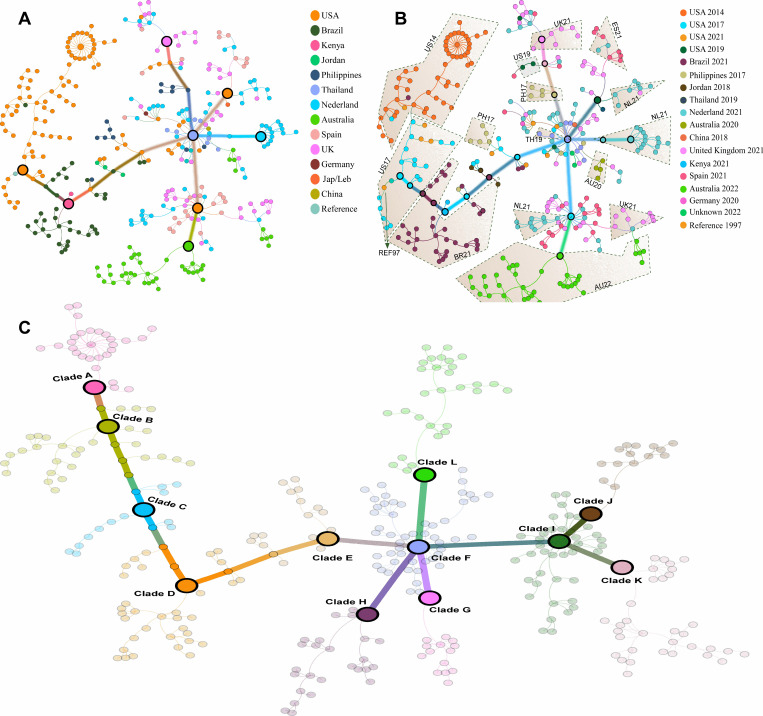
Transmission distribution of the RSV genomes. (A), Country wise network distribution of the RSV genomes derived through effective parsimony informative sites (ePIS). (B), Country and year wise network of complete RSV genomes. (C), calculated transmission patterns via community detection by modularity approach.

To trace the transmission routes, and contribution, we mapped 474 RSV genomes on the viral evolution network. Our data indicated individual clusters such as cluster of the USA 2014 sequences, connected to cluster of the USA 2017, which further connected the Brazilian cluster 2021. In contrast, individual sequences from Jordan 2018 and the Philippines 2017 were located at the boundaries of the Brazilian cluster, without forming distinct clusters of their own. Our analysis revealed that samples from North and South America formed distinct clusters, separate from those of the European samples. However, genomes reported from the USA 2019 were found mixed with genomes reported from Europe (Fig 1A).

To understand the transmission pattern, we used a network modularity function and clustered the whole network agnostic of country and date of collection. We retrieved 12 clades (clade A to clade L). Interestingly, our data predicted a unique pattern, for instance terminal clade A, containing purely genomes reported from the USA in the year 2014, which connected clade B retaining genomes reported from the USA in the year 2014 and 2017. Further, clade C, a direct descendent of clade B, contains all the genomes reported in 2017, except one sequence from the USA 2021. Apart from few genomes reported from the USA 2017, Philippines 2017 and Jordan 2018, clade D contain > 95% of the genomes reported from Brazil 2021. To our interest the smaller clade E, contains representation of almost all the previous clades and connects the bigger node clade F. All the previous clades did not show a single genome reported from the Europe therefore, for better understanding, we call clade F, a European clade, as it is the first with European representation that also gave birth to diverse nodes reported from European countries (Fig 1C).

The European node (clade F), is tetra-furcated into clade G, H, I and L. Among them, clade H contained samples of Nederland, Spain and the United Kingdom reported in 2021 while clade G was found enriched with all the sequences reported from Nederland 2021. Likewise, clade L is formed from the genomes reported in the United Kingdom 2021, the USA 2019, and two genomes from Spain 2021. Interestingly, clade L emerged from the European cluster through genomes reported from Philippines 2017 and the USA 2019. Finally, Clade I that contains genomes from Spain, Nederland and the United Kingdom 2021 which is bifurcated to clade J and K, where clade J contains samples from the United Kingdom 2021 that leads to Australia 2022 and the clade K only contains all the genomes reported from Australia reported in 2022. All the samples are mapped with country, year and transmission wise in (Fig 1B and 1C).

Collectively, these analyses indicate the global prevalence and the presence of different RSV type A strains. For instance, genomes reported from Australia in 2022 were gathered in two different clusters emerging from the European genomes, suggesting the presence of two different strains. However, genomes reported from Australia in 2020, are lying distantly for those reported in 2022. Likewise, the genomes from the UK reported in 2021, were found almost everywhere with European genomes, depicting the possibility of numerous RSV strains. Genomes from Spain and the Netherlands were also found in 2 and 3 different nodes, highlighting the circulation of more than one strain in that country.

### Mutation and substitution frequencies of synonymous and non-synonymous sites in RSV genomes

According to the NCBI records, RSV contains ten to eleven protein coding genes, i.e., non-structural protein 1 and 2 (NS1 and NS2), nucleoprotein (N), phosphoprotein (P), matrix 1 protein (M), small hydrophobic protein (SH), attachment protein (G), fusion protein (F), matrix 2 protein (M2), and polymerase (L). We calculated substitution observed in RSV genomes for all coding sequences (CDS) of all ten genes, particularly synonymous and non-synonymous substitutions. All the substitutions were accessed and calculated against reference strain U39662.1. A total of 6257 (45%) sites were observed in substitutions, and among them 2099 (15.1%), 3027 (21.7%), 611 (4.3%), and 473 (3.4%) were synonymous, non-synonymous, both and terminations, respectively. Among the non-synonymous substitutions, 1442 (10%) sites were found to have changes in amino acid properties. The highest termination substitutions were found in L followed by N and G proteins (Table 1).

**Table 1:** Statistics of all types of substitutions observed in individual genes of RSV.

Gene	Length (CDS)	Proportion of Mutation sites	S(p)	N(p)	S-N(p)	Termination(p)
**NS1**	420	105 (25.0%)	79 (17.3%)	29 (6.9%)	3 (0.7%)	0
**NS2**	503	110 (21.8)	75 (14.9%)	30 (5.9%)	5 (0.9%)	0
**N**	1176	554 (47.1%)	200 (17.0%)	253 (21.5%)	67 (5.6%)	34 (2.8%)
**P**	726	214 (29.4%)	138 (19.0%)	67 (9.2%)	9 (1.2%)	0
**M**	771	177 (22.9%)	138 (17.8%)	33 (4.2%)	6 (0.7%)	0
**SH**	195	63 (32.3%)	33 (16.9%)	25 (12.8%)	4 (2.0%)	1 (0.5%)
**G**	897	496 (55.2%)	162 (18.0%)	298 (33.2%)	28 (3.1%)	8 (0.8%)
**F**	1725	468 (27.1%)	349 (20.2%)	106 (6.1%)	13 (0.7%)	
**M2**	585	150 (25.6%)	94 (16.0%)	47 (8.0%)	8 (1.3%)	1 (0.1%)
**L**	6498	3920 (60.32%)	831 (12.7%)	2139 (32.9%)	468 (7.2)	432 (6.6%)
**Complete**	13892	6257 (45.0%)	2099 (15.1%)	3027 (21.7%)	611 (4.3%)	476 (3.4%)

In protein L and G, we also observed higher and similar non-synonymous mutation frequency compared to all other genes of RSV. However, F and P proteins have relatively shown a higher number of synonymous substitutions than non-synonymous. Complete details for substitutions and substitution type for all individual genes can be accessed in form (S1 Table).

**Fig 2:**
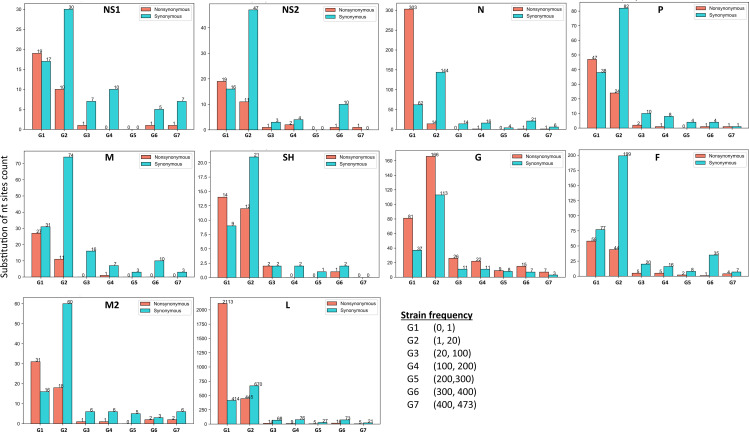
Overall substitution frequencies of all coding genes coded by the RSV genome. The Frequencies of only synonymous and non-synonymous substitutions are shown here. Y-axis represents the number of substitutions, and the X-axis depicts the number of genomes or samples participated.

To evaluate the overall substitution frequency of the mutated sites, present in all ten CDSs, we binned the substitution frequency into seven different groups (G1 to G7) and depicted the frequency distribution of 5126 substituted sites (3027 non-synonymous and 2097 synonymous) of the 474 sequenced genomes. The group number on the X-axis indicates the number of strains participating in a substitution event at a particular site, whereas the Y-axis shows the number of substitutions in a respective CDS (Fig 2).

Our results indicated that apart from the initial groups, non-synonymous mutations were found relatively higher than the distribution of synonymous mutations. Comparing all the CDSs, the CDS of G protein showed the highest number of non-synonymous mutations where 53 mutations were found in G4 - G7, meaning the participation of 200 samples. We have also observed that CDSs of NS1, NS2, SH, and M proteins offered relatively lower sites for non-synonymous mutations in the majority of the sequenced RSV strains. The distribution of substitution frequency of each codon in each gene can be found in the supplementary S1 Table.

Overall, these analyses provide complete mutational information of all ten RSV’s CDSs. Such key features can also be used to assess the evolutionary pressure on selected sites or response to therapeutic agents.

### Effects of mutation and evolutionary dynamics

The investigation of evolutionary metrics such as nucleotide diversity (π), Tajima’s D, Shannon entropy, and dN/dS is critical for elucidating the mechanisms underlying genetic variation, selective pressures, and evolutionary dynamics. Each of these metrics offers distinct insights into the evolutionary processes shaping viral genomes. Our findings revealed significantly elevated nucleotide diversity (π = 0.049) in the RSV G glycoprotein gene compared to other genes, which exhibited values ranging from π = 0.017 to 0.022. This observation was further corroborated by Shannon entropy analysis, which demonstrated an average entropy value of 0.129 for the G glycoprotein gene, indicating higher variability across the RSV genome. In contrast, the remaining genes displayed average entropy values within the range of 0.0144 to 0.0160, suggesting relatively conserved regions.

**Fig 3:**
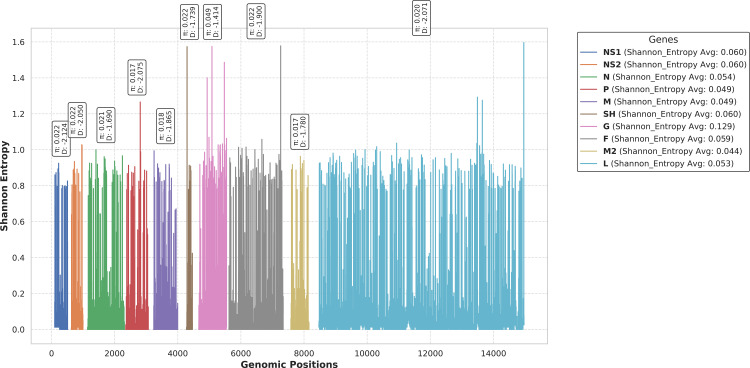
Statistical analysis of RSV evolution. The plot illustrates the Shannon Entropy values for each gene in the RSV genome, with genomic positions on the X-axis and entropy values on the Y-axis. Each gene is represented by a solid line and different color, and the corresponding Nucleotide Diversity (π) and Tajima’s D values are displayed vertically above each gene’s plot. Genomic boundaries for every gene are used from the reference RSV genome NC_001803.1. The legend, positioned outside the figure, includes the gene names in bold and their average Shannon Entropy values. The plot highlights the variability and evolutionary dynamics across the RSV genome.

Subsequent analysis using Tajima’s D indicated negative values across all RSV genes, consistent with the presence of subpopulations. This outcome was anticipated, as the data set comprised samples from diverse geographical regions, reflecting population structure and potential demographic influences (Fig 3).

To assess selective pressures acting on individual genes, we computed the dN/dS ratio, which quantifies the rate of nonsynonymous to synonymous substitutions. Our results identified codons under positive selection in nearly all genes, with the G and L genes exhibiting particularly strong selective pressures. Notably, codon 115, 286, and 290 in the G gene, along with codon 1182 in the L gene, displayed dN/dS values exceeding value of 50 (S2 Table and S1 Fig), highlighting their potential roles in adaptive evolution.

In summary, these comprehensive analyses underscore the pivotal role of the G glycoprotein gene in driving RSV evolution and transmission. The elevated nucleotide diversity, Shannon entropy, and positive selection observed in this gene suggest its significant contribution to viral adaptation and host interactions.

### Mutation frequencies in non-coding regions of RSV genomes

RNA viruses, such as Respiratory Syncytial Virus (RSV), are characterized by high mutation rates, which play a critical role in their evolutionary adaptability and survival [21]. Leveraging the RSV reference genome (NC_001803), we investigated the mutational patterns within non-coding regions, with a particular focus on RNA-coding segments. According to the genomic annotations of the reference sequence, three miscellaneous RNA (misc_RNA) genes were identified, corresponding to gene IDs 1724895, 1494476, and 1724894. These genes span nucleotide positions 1 to 44 (1724895), 8460 to 8527 (1494476), and 15038 to 15191 (1724894), respectively.

**Fig 4:**
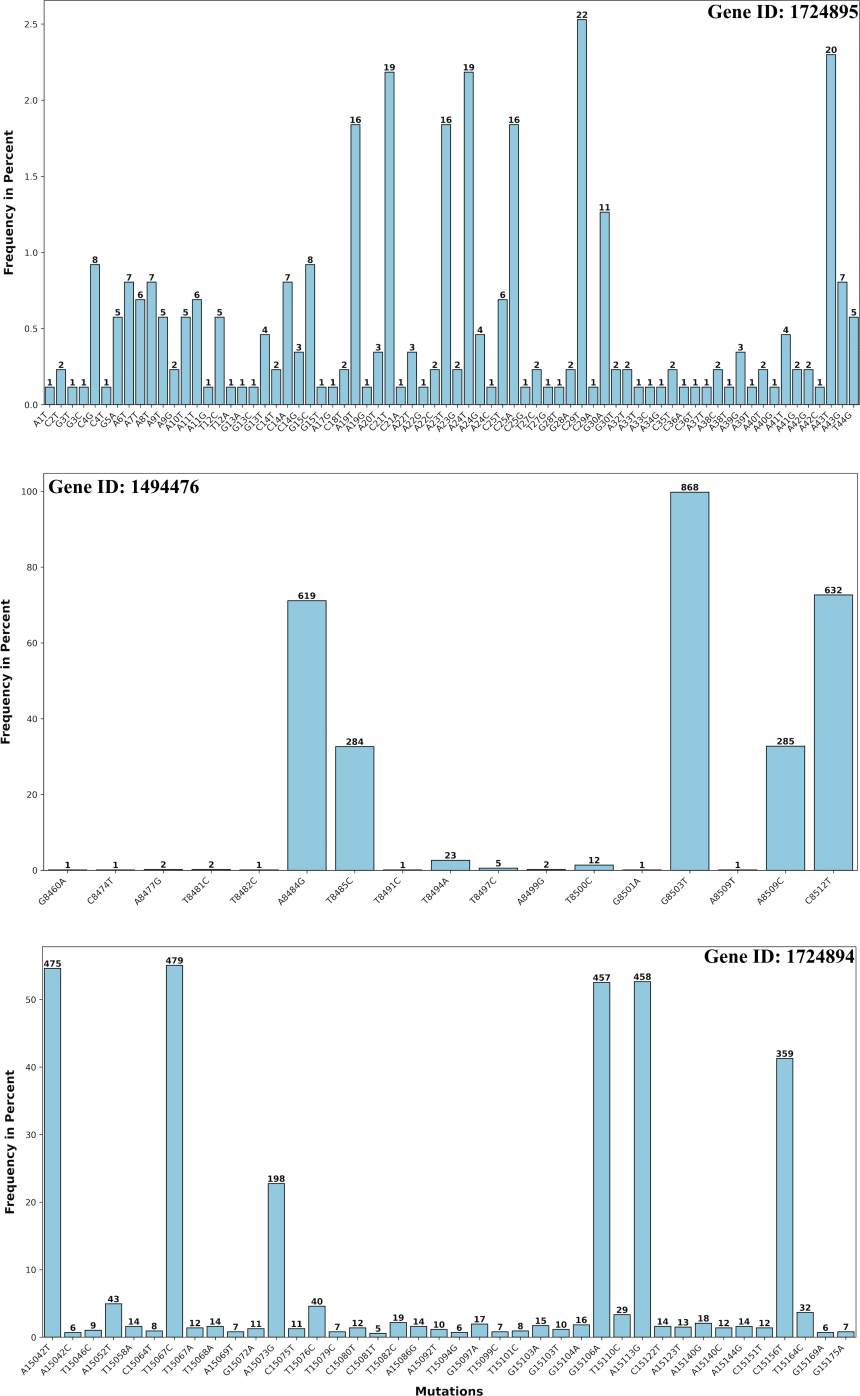
Nucleotide substitution frequencies of RSV non-coding segments. The analysis plot depicts the nucleotide substitution frequencies for the RSV non-coding segments (misc_RNA genes 1724895, 1494476, and 1724894), revealing a variable range of substitution frequencies across different genomic sites. The plot highlights the heterogeneity in mutation patterns within these non-coding regions, providing insights into their evolutionary dynamics and potential functional roles.

Our analysis revealed a higher mutational propensity in the downstream-located gene 1724894 compared to genes 1724895 and 1494476. In contrast, the mutation frequencies in the extreme upstream and downstream regions were relatively low, ranging from 2% to 8%. Notably, four specific positions within gene 1494476; A8484G, T8485C, A8509C, and G8512T exhibited significantly higher mutation prevalence, with frequencies exceeding 30% (Fig 4). These findings highlight distinct mutational hotspots within the RSV genome, particularly in the non-coding RNA regions, which may contribute to the virus’s adaptive mechanisms.

### Substitution hotspots in RSV type A genomes

**Fig 5:**
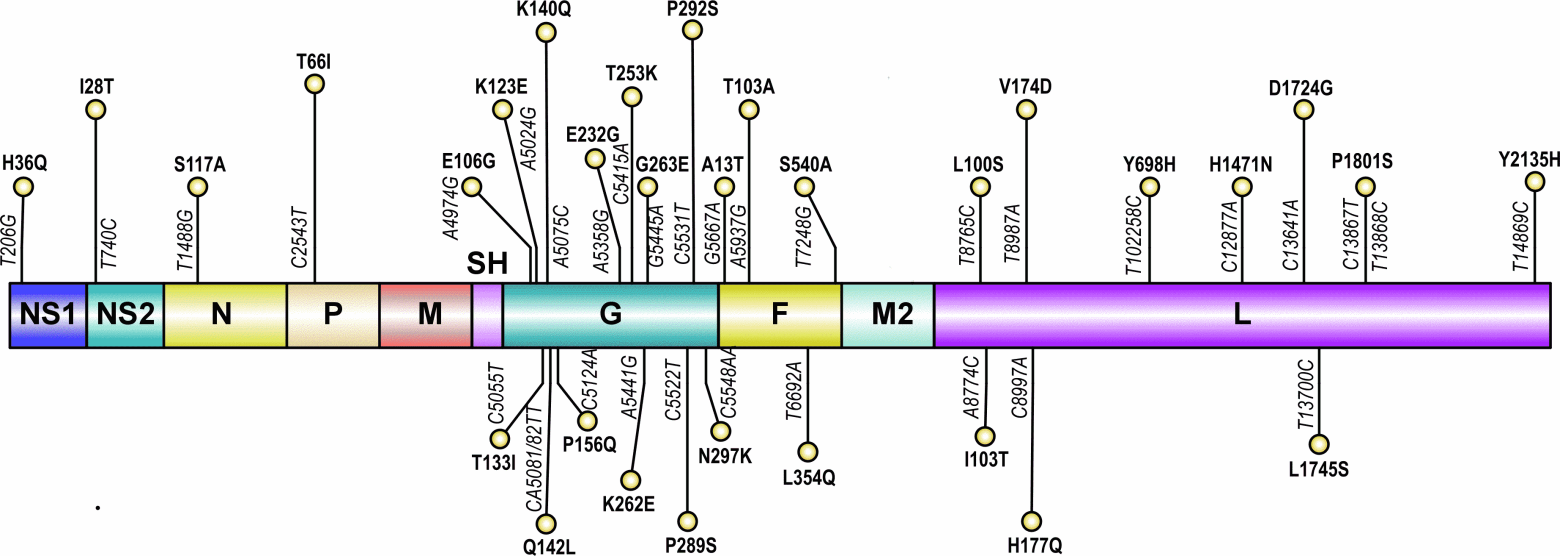
Schematic Presentation of hotspots mutations in RSV proteins. The nucleotide positions are numbered according to reference genome U39662.1 while the proteins are numbered as per protein.

Next, we were interested in identifying the hot-spot substitution sites in RSV CDSs. Similar to the analysis [18] for SARS-CoV-2, we defined a criterion for hotspot regions. A site with a substitution frequency over 200 will be considered a hot-spot site, or a site that offered substitution to more than 200 RSV strains (42% in our case) will be considered a potential substitution hot-spot. Second, the respective site must allow non-synonymous substitution, and the observed amino acid should record a change in amino acid properties. A total of 367 substitution sites were found with > 200 substitution frequency where 290, and 77 were synonymous and non-synonymous respectively. Among 77 non-synonymous substitution sites, 31 were those affecting amino acid properties (either changing polarity or charge difference) considered hot-spot substitution sites. These 31 hotspots were found distributed across F protein (4), G protein (13), L protein (10), N1, N2, P, and N (1 each) (Fig 5). Among them, one of these hotspots that resulted in CAA to TTA (Q142L), and CAA to TCA (Q142S) in G protein offered double substitutions.

## Discussion

Respiratory syncytial virus (RSV) is a communal respiratory virus that can cause mild to severe medical conditions, predominantly in young children, elderly adults, and people with certain chronic medical conditions. In some cases, it can lead to more serious illnesses such as bronchiolitis or pneumonia. Here, we detected a probable transmission pattern based on genetic global variability observed in RSV genome types A that in turn may also explain the classification of different strains circulating worldwide. The RSV evolutionary trajectories and geographical distribution are highlighted through network community clustering across different countries. Studying viral genetic variability is of utmost importance as it informs on the emergence of new escape variants and strains also deduce circulation patterns [22]. The results obtained in the current analysis are in line with the previous studies, suggesting RSV heterogeneity at both regional and temporal levels [23]. The tight clustering of the USA sequences in 2014 and 2017, which is diverged into Brazilian cluster in 2021, observed in the current analysis, depicts that viral strains may persist and evolve locally for a period before spreading to other regions. These findings are in agreement with previous reports conducted on global epidemiology of antigenically distinct viral strains of RSV, indicated a seasonally favored outbreak, dominating different regions at different times [24,25]. There are reports claiming simultaneous circulation of RSV in different communities [26]. However, in a particular region, strains of one group often dominate for one or more continuous seasons. For instance, in the year 1988 to 1990, several European countries were faced RSV epidemics caused by GA1 lineage [25]. Subsequently, in the following years RSV GA1 strains were detected less frequently in these countries. It also suggests that genetically diverse RSV strains circulate simultaneously in a locality [13]. Thus, certain RSV strains may have wide geographic dissemination and that the observed variability is predominantly temporal rather than geographic.

In viral genomes, the presence of synonymous and non-synonymous mutations with varying frequencies are primarily based on evolutionary pressure. It has been reported that in many viruses, the accumulation of non-synonymous mutations is higher compared to synonymous and is partly due to its role in immune evasion [27]. Similar results were observed in the current study where non-synonymous mutations were found partly higher (21.7%) than synonymous mutations (15.1%). Also observed mutation patterns of SARS-CoV-2 depicted higher non-synonymous mutations and were attributed to evasions schemes of host immune system [27–29].

The role of misc_RNA segments in RSV (Respiratory Syncytial Virus) genomes primarily involves the regulation of viral replication and interaction with host cells. These non-coding RNA segments play significant roles in modulating host-virus interactions and potentially influencing the virus’s ability to establish infections. Various host-derived non-coding RNAs, such as miRNAs, lncRNAs, and tRNA-derived RNA fragments, are implicated in RSV infections by regulating gene and protein expression, impacting the disease mechanisms of RSV and potentially serving as biomarkers for diagnosis and targets for antiviral therapies [30,31]. Particularly, in the context of plant RNA viruses like Rice stripe virus (RSV), variations in the 3’-terminal regions of the viral genome, influenced by host alternations, play a role in viral adaptation and replication. Such variations can affect the virus’s ability replicating in different hosts, such as plants and insect vectors [32].

RSV is an enveloped virus with a linear, single-stranded, negative-sense RNA genome, belonging to the *Paramyxoviridae* genus, and *Pneumoviridae* family. It retains two antigenic groups of strains, A and B, and multiple genotypes within the two groups. Structurally, RSV consists of ten genes, encoding 11 proteins. Of these eight are structural including the glycoprotein G, the fusion protein F and the hydrophobic SH protein. On the inner side of the envelope, a non-glycosylated matrix protein M is present. There are also four nucleocapsid proteins that include the nucleoprotein N, the phosphoprotein P, the transcription factor M2-1, and the large subunit of polymerase L. [33,34]. The proteins G, L and F are amongst the key proteins involved in the viral entry into the host, virus replication and immune system evasion [35].

In particular, protein G and F proteins are considered important because both can induce neutralizing antibodies, and are heavily glycosylated, which has been shown to affect with antibody recognition [36–38]. Structurally, the G protein comprises three domains: a cytoplasmic domain (1–37 amino acids), a transmembrane domain (38–66 amino acids), and an ectodomain region (67–312) [39,40]. Interestingly, all the hotspot positions we identified belong to the ectodomain of the G protein. There are individual reports in G, F, and L proteins [41–46] and we believe that this report will assist researchers to directly pick the hotspot regions for biochemical testing.

The dN/dS ratio has been studied for RSV to assess selective pressure on genes. A study on the genetic variability of the G protein gene among RSV isolates from India found that the dN/dS ratio was higher between the GA2 and GA5 genotypes (1.78), indicating greater selective pressure, compared to within the genotypes (0.69) [47]. In our case, higher nucleotide diversity in the RSV G glycoprotein gene compared to other genes was observed.

Overall, these analyses provide a complete picture of the RSV genomes, their mutations, transmission probability across the globe, and codon selective pressure analysis.

## Conclusion

In conclusion, this study provides a comprehensive analysis of RSV evolution and transmission dynamics through a multi-faceted approach. By employing transmission network analysis using an effective parsimony method, we identified key transmission clusters and patterns, shedding light on the spread of RSV across populations. Additionally, the identification of hotspot regions within the genome highlighted areas of heightened variability and potential functional significance. Evolutionary analyses, including nucleotide diversity, Shannon entropy, Tajima’s D, and dN/dS ratios, revealed distinct selective pressures acting on RSV genes, with the G glycoprotein gene emerging as a major driver of viral evolution. Furthermore, the inclusion of misc_RNA genes in our analyses provided novel insights into the role of non-coding regions in RSV diversity. Collectively, these findings enhance our understanding of RSV epidemiology, evolution, and adaptation, offering valuable insights for future surveillance, therapeutic development, and vaccine design.

## Supporting information

S1 Table Complete details of the substitution in each coding gene of the RSV type A. While studying this data, please take care of the INDEL event. After the INDEL event normally codon numbers of changes.
(XLSX)

S2 Table Codon-wise selective selection details for all the RSV genes. (XLSX)

S1 Fig Visualization of codon under positive selection of all the RSV genes. (TIFF)
